# The state of sleep and the current brain paradigm

**DOI:** 10.3389/fnsys.2015.00139

**Published:** 2015-10-13

**Authors:** Ivan N. Pigarev, Marina L. Pigareva

**Affiliations:** ^1^Laboratory of Sensory Information Processing, Institute for Information Transmission Problems (Kharkevich Institute), Russian Academy of SciencesMoscow, Russia; ^2^Laboratory of Neuroontogenesis, Institute of Higher Nervous Activity and Neurophysiology, Russian Academy of SciencesMoscow, Russia

**Keywords:** sleep, cerebral cortex, slow wave sleep, sleep function, visceral control

## Abstract

Up to the present time cerebral cortex was considered as substrate for realization of the highest psychical functions including consciousness. Cortical sensory areas were regarded as structures specialized for processing of information coming from one particular modality (visual, auditory, somatosensory, and so on). However, studies of cortical activity in sleep-wake cycle demonstrated that during sleep the same neurons in the same cortical areas switch to processing of signals coming from the various visceral systems. After awakening these visceral responses disappear and the neurons return to processing of the information coming from the exteroreceptors. These observations indicate that most likely cortical areas are universal processors, which perform particular operations with incoming information independent of its origin. During wakefulness, results of the information processing on the cortical level should be directed to structures connected with organization of behavior and consciousness, while during sleep cortical outputs should be redirected to structures performing integration of the visceral information. Thus, results of sleep studies indicate that current brain paradigm should be changed.

In spite of thousands of studies devoted to the investigation of various aspects of sleep, up to the present time there were no generally accepted hypothesis, which could unite all reported observations in a single and non-contradictive theory. This fact is reflected in the main paradox of sleep state. It is recognized that sleep deprivation causes severe visceral dysfunctions and finally unavoidable death of animals without obvious pathological alterations in the brain morphology. On the other hand the brightest and the most studied events during transition from wakefulness to sleep take place in the central nervous system (including cerebral cortex). It remains unclear how all known changes in cortical activity during sleep could be related to the visceral health? This situation let us propose that difficulties with understanding of sleep function could be connected with the wrong general paradigm, as it was defined by Kuhn (1962). Some considerations included in this article were presented in the lecture, which was given by Pigarev (2013) on the workshop “Sleep: a window to the world of wakefulness,” in Rostov-on-Don, Russia.

The current paradigm of brain organization was established in the twentieth century and was based on the results obtained in studies performed mainly in wakefulness. Below we formulate some elements of this paradigm as we see it.

1. The cerebral cortex is the highest level of information processing. Cognitive functions and consciousness are primarily associated with activity of the cortical neurons.
2. Visceral organs are working under the control of the autonomous nervous system and have minimal, if any, representation in the cerebral cortex.

Within the frame of this paradigm it was quite natural to consider sleep as “the state for the brain,” and it was not surprising that most of sleep studies were addressed to its impact to cognitive functions and memory. At the same time, there were no place for mechanisms, which could link sleep and the visceral health. However, some results do not fit to the presented paradigm. First of all the general level of neuronal activity in the cerebral cortex, estimated in electrophysiological studies, is practically the same in wakefulness and during sleep. If activity of the cerebral cortex is indeed related to consciousness one will expect that during sleep, when consciousness is often totally disengaged cortical neuronal activity will be reduced. Stability of the mean level of cortical activation during transition from wakefulness to sleep was confirmed in studies using neuroimaging methods (e.g., Balkin et al., 2002), and these authors also paid attention to this obvious contradiction.

There was an attempt to bypass the fact of equal cortical neuronal activation during wakefulness and slow wave sleep, when consciousness is strongly reduced. So, Tononi (2005) offered the information integration theory of consciousness. This theory mainly linked consciousness with activity in thalamo-cortical network, and proposed that not the mean level of neuronal activity in the cerebral cortex, but rather integration of this activity in various brain regions is important for consciousness. Some experiments with transcranial magnetic stimulation do demonstrated, in human subjects, reduced number of the cortical areas activated in response to such direct stimulation of the cortex during sleep (Massimini et al., 2010). However, earlier experiments in animals (Berlucchi et al., 1967) demonstrated strong reduction of callosal connectivity during REM sleep—the period when information integration theory predicted increase of transcortical integration of information because of dreaming recognized by this theory as indication of consciousness emerging during sleep (Siclari et al., 2013).

In parallel with the attempts to stay within the traditional paradigm, more and more scientists associated the highest brain functions not with the cortical activity but with the “supra-cortical” levels of information processing, for example the basal ganglia (e.g., Crick and Koch, 2005; Stocco et al., 2010; Koubeissi et al., 2014; Stiefel et al., 2014).

In addition, within the current paradigm cortical areas were regarded as structures specialized for processing of information coming from one particular sensory modality (visual, auditory, somatosensory, and so on). However, it was shown that redirection of visual cortical fibers to auditory pathway makes neurons in auditory area responsive to visual stimulation, and even more, they demonstrated features typical for the visual cortex like direction and orientation selectivity (Sur et al., 1988). And recently it was shown that in particular conditions neurons even in the most “specific” primary visual area could start responding to somatosensory stimulation (Saleem et al., 2013).

History of the computational science showed that development of automatic devices specialized for a single function was not an efficient way, and all modern computers are based on the universal processors. It was possible to think that probably cortical areas also are universal processors tuned not so much for particular sensory modality, but rather for particular algorithms of incoming information processing.

Taking this into account we decided not to restrict ourselves by the frame of the abovementioned paradigm in attempts to understand the function of sleep. The first target for revision was status of the cortical sensory areas.

We hypothesized that the same cortical neurons, which process exteroceptive information in wakefulness, switch to the processing of visceral information during sleep (Pigarev, 1994, 2014). In according to this proposal namely visceral inputs determine high level of activation in the cerebral cortex during sleep. Periodic pattern of visceral afferent flow coming from gastro-intestinal, cardiovascular and respiratory systems could define periodic burst-pause neuronal activity and slow waves in cortical EEG during sleep.

Experiments, which were undertaken in order to check the abovementioned proposal, are reviewed in several recent articles (Pigarev and Pigareva, 2012, 2014; Pigarev, 2014). The results of these experiments can be divided into three groups. First of all, it was demonstrated that indeed neurons and cortical evoked responses in visual and somatosensory cortical areas of cats, rabbits and monkeys, begin responding to electrical or magnetic stimulation of stomach and intestine during sleep. In all cases these visceral responses disappeared immediately after awakening of the animal.

In the second group of studies non-natural electrical or magnetic stimuli were excluded. Instead of that natural myoelectrical activity of stomach and duodenum was recorded together with neuronal activity in various visual cortical areas. It was shown that during slow wave sleep cortical neurons and duodenum established causally defined correlation of activity, and cortical neurons appeared to be selective for particular types of duodenal waves (Pigarev et al., 2013). It was shown that periods of short desynchronization of the cortical EEG, well known during slow wave sleep, often coincided in time with migrating myoelectrical complexes of stomach (Figure 5 in Pigarev and Pigareva, 2014).

In the third group of experiments it was demonstrated that changes of the content of the gastric cavity during stable slow wave sleep lead to tonic and long lasting changes of neuronal firing in the cerebral cortex revealed by Fano factor analysis (Pigarev et al., 2014). In these experiments the influence on interoreceptors was achieved by injection of small amount of warm water or solution of Loperamide into the stomach through the chronically implanted fistula.

In pilot studies visceral responses in cortical sensory areas during sleep were obtained not only in response to particular events in gastro-intestinal system, but also to heart activity and respiration (Figure 6 in Pigarev and Pigareva, 2014).

Performed experiments confirmed the proposal that cerebral cortex indeed switches to the processing of visceral information during sleep.

One often argues that visceral signals can reach the cortical level in both awake and sleep states of the animal. During wakefulness these signals are masked by the more intensive flow of exteroceptive afferentation. This hardly can be so. Such masking of the sensory signals do occurs at the very first levels of information processing and is connected with the work of mechanisms of adaptation. At the cortical level sensory signals are coming already within the ranges optimal for the further processing. And indeed in some our experiments (Pigarev, 1994) when the cats were woken during deep sleep but were still drowsy, responses to visceral stimulation were recorded, together with visual responses of the neurons. However, these visceral responses disappeared as soon as the cats became more awake. At the same time no detectable changes in the visual responses could be noticed. That is why it seems reasonable to expect the existence of special structures with a gate function, which block access of interoceptive signals to the cerebral cortex in wakefulness, and access of exteroceptive signals during sleep. The work of such gating mechanism for visual afferentation was demonstrated in several studies at the level of thalamus (e.g., Mukhametov and Rizzolatti, 1970).

One may ask how visceral information can reach those cortical areas, which in wakefulness process extero and proprioceptive information. This was investigated in case of somatosensory areas. Starting from the middle of twentieth century evoked responses to stimulation of vagus or splanchnic nerves were demonstrated in various cortical areas in experiments under anesthesia (e.g., Amassian, 1951; Downman, 1951; Ito and Craig, 2003). However, in experiments without anesthesia these responses could not be reproduced. In wakefulness neurons of these areas responded to somatosensory or visual stimulation. Previously observed visceral responses were considered as artifacts of anesthesia. Now it became clear that visceral projections to these cortical areas do exist but they are activated only during sleep of these areas.

It was also well known since nineteenth century that visceral and somatosensory afferents terminate at the same neurons in the spinal cord, and thus visceral information may travel to the cerebral cortex by the fibers of somatosensory columns. The fact of such combined projections was confirmed also in many later studies (e.g., Cervero et al., 1984), and this overlap was regarded as the most probable mechanism of referred pains (e.g., Hobson et al., 2010). However, this overlap created the unresolved problem—how the central nervous system manages to distinguish what kind of information is coming if the same neuronal fibers transferred this information. Visceral theory of sleep offers solution of this problem—transmission of the somatosensory information happens during wakefulness, while visceral information travel to the central nervous system using the same fibers but during sleep when movements are excluded and muscles are relaxed.

Concerning pathways of the visceral information to the visual cortical areas we can offer only some considerations. It is known that in the main thalamic visual relay—lateral geniculate nucleus, retinal synaptic terminals form only one third. Another one third of terminals belong to backward cortico-thalamic projections. And the remaining third is of non-visual origin, and comes from the pontine and brain stem regions (Hughes and Mullikin, 1984). Activation from these pontine nuclei reaches lateral geniculate nucleus and cortical visual areas during sleep, especially during REM sleep, and is responsible for the well-known ponto-geniculo-occipital waves (Brooks and Bizzi, 1963). The primary origin and meaning of this pontine activity were not investigated. However, location of those nuclei let propose their visceral nature. Other brain stem projections to the lateral geniculate nucleus also can be engaged into the transfer of the visceral information.

Observed transition of the cerebral cortex to processing of the visceral signals during sleep on our opinion is a strong argument in favor of revision of the current brain paradigm. Sleep studies are not alone on this way. Many other experimental results in various fields of neuroscience and clinical observations also indicate that existed brain paradigm needs revision (Möller, 2013).

Below we offer some features of the emerging brain paradigm.

1. Cognitive functions and consciousness are connected not with the cortical activity but with the supra-cortical levels of information processing located in subcortical structures.
2. Visceral organs are indeed under the control of the autonomous nervous system but only during wakefulness. All areas of the cerebral cortex and probably other brain structures become visceral during sleep.

Analysis of the history of science development led the founder of the science paradigm ideology Kuhn (1962) to very sorrowful conclusion. He wrote that any new scientific paradigms become considered only by the next generation of scientists. So, we are looking with hope on young scientists coming to work in System Neuroscience.

## Conflict of interest statement

The authors declare that the research was conducted in the absence of any commercial or financial relationships that could be construed as a potential conflict of interest.
